# Testosterone dynamics of migratory birds during stopover

**DOI:** 10.1038/s41598-025-95413-z

**Published:** 2025-04-06

**Authors:** Armando Alberto Aispuro, Ivan Maggini, Leonida Fusani, Virginie Canoine

**Affiliations:** 1 Department of Interdisciplinary Life Sciences, Konrad Lorenz Institute of Ethology, University of Veterinary Medicine, Vienna, 1160 Vienna, Austria; 2 Coal Oil Point Reserve, University of California, Santa Barbara, CA 93106 USA; 3 Department of Behavioural and Cognitive Biology, University of Vienna, 1090 Vienna, Austria

**Keywords:** Body condition, Hormones, Migration, Sahara Desert, Simulated territory intrusion, Stopover territoriality, Animal migration, Ecophysiology, Animal physiology, Animal behaviour, Gonadal hormones

## Abstract

Birds migrating in the spring must balance energy with hormonal preparations in anticipation of the forthcoming breeding season. We investigated the relationships between testosterone, body condition, sociality, territoriality and fueling rates in Western Subalpine Warbler (*Curruca iberiae*) males during a trans-Saharan stopover. Baseline testosterone was highly variable in correspondence with the transitional nature of spring stopover. Some individuals reached breeding testosterone levels while others had undetectable levels. Testosterone varied with body condition suggesting an endocrine-energy link during migration. Simulated territory intrusions induced an increase of testosterone up to physiological maxima- a similar pattern to breeding contexts. Testosterone was negatively associated with territorial male density, suggesting a ‘dear enemy’ effect related to the daily variation in social stability. In repeatedly-sampled individuals, stopover duration and fueling rate were not correlated with baseline testosterone. However, as testosterone decreased, body condition increased. This suggests that stopover territoriality may reduce the reported negative effects of chronically high testosterone. Our data supports the hypothesis that hormonal preparation for breeding may already occur during stopover, and that this is largely linked to body condition. In this system, the endocrine-energy relationship is likely maintained by stopover territoriality. We conclude that male-male social contexts are modulated in similar ways during spring migration as during the breeding life history stage.

## Introduction

In birds, spring migration immediately precedes the all-consequential breeding life history stage. Therefore, migratory birds are tasked with transitioning towards the breeding phenotype, all while enduring the physiologically taxing demands and peril of traveling great distances^1^. Migratory stopover, where birds stop to rest and forage between flights, offers a unique platform to examine the relationships among the overwintering, migratory and breeding life history stages- and their associated physical, hormonal and behavioral attributes^2^. Therefore, the migratory period, and specifically stopover, may be key in understanding the overlap and transitioning among life history stages^3–6^.

Little is known about how hormonal activity relates to migratory stopover behavior, duration and energetics in free-roaming songbirds. Additionally, it is yet to be explored if life history benefits from activities at stopover are gained on an intra-individual level (i.e., measured through repeated samples over time). One such benefit of stopover stays may be gradual physical preparations in anticipation of the breeding stage^1,4^. While migratory flight may be composed of haphazard and unpredictable events, stopover is a relatively controlled and stable component within migration. We therefore explore the idea of stopover as a platform for key life history changes to occur.

Recently, we found that Western Subalpine Warbler (*Curruca iberiae;*‘Western’ is hereafter omitted) males established defined territories, defended them against conspecifics, and reinforced boundaries by singing during prolonged stopovers in the Sahara Desert^2^. However, fueling did not appear to be related to aggressive territoriality, as female Subalpine Warblers and other species had similar fueling rates but were not territorial. Because birds sing and defend territories, we speculated that some ‘breeding-like’ characteristics were being expressed, while some migratory characteristics were being suppressed (i.e., refueling).

The steroid androgen hormone testosterone is often implicated as a primary agent influencing or modulating aggressive and reproductive behaviors in birds^1,7^. Generally, in birds breeding in temperate zones, baseline circulating levels of testosterone peak at the onset of breeding activities and are lowest during the non-breeding season^8–10^. Territorial aggression, which peaks during the reproductive stage, is also largely regulated by the activity of testosterone^10–14^. Seasonal reductions in testosterone (i.e., during non-breeding periods) is perhaps a strategy to reduce metabolic, fitness and survival costs associated with persistently high testosterone and aggression^15–17^. These predictable seasonal changes have allowed researchers to use testosterone level as a relative indicator of an individual’s position along a life history gradient, such as from the non-breeding season to the breeding season^6,18^. Indeed, there is growing information on how testosterone may affect, or is affected by long-distance migration (e.g., refs 19 and 20). Here we focus on stopover and how the activities and dynamic events that occur during this period relate to testosterone.

We examined three separate components relating to the action of testosterone in birds during a spring migratory stopover. The first component is a descriptive exploration of testosterone at stopover, and the second two are hypotheses. Respectively, these are 1) Baseline Testosterone at Stopover (BTS), 2) Stopover Challenge Hypothesis (SCH) and 3) Hormonal Staging Hypothesis (HSH).

First, in BTS, we investigated how physiological, temporal and social parameters are related to baseline testosterone levels of free-roaming males. We predicted that baseline testosterone levels should be positively associated with the season (e.g., from increasing photostimulation through day length (e.g., refs^19,20^), with body condition (e.g., ref^21^) and with territorial male density on the day individuals were sampled (e.g., refs^12,22^). Additional effects may come from age and the time of day they were sampled.

Secondly, we tested if aggression experienced during migratory stopover is followed by increases in testosterone concentrations- as commonly observed in male-male breeding contexts^7^. The SCH portion of this study refers to one prominent prediction and component within Wingfield et al.’s original Challenge Hypothesis^7^ but distinguishing ours as occurring explicitly outside of a breeding context and during migratory stopover. Accordingly, we predicted that males showing territorial behavior during stopover would respond to simulated territory intrusions by elevating their levels of testosterone relative to stopover baseline levels. We contextualized this by comparing levels following a simulated territory intrusion to physiological maximal levels at stopover as estimated by pharmacological stimulation.

Finally, under the HSH we tested the hypothesis that birds use stopover sites as platforms to advance their life history position towards a breeding phenotype. We made two predictions under the HSH. First, we predicted that birds at stopover will have similar testosterone levels as birds at breeding sites, reflecting the overlap among life history stages^1,18^. This may support a link between hormonal staging and stopover territoriality. In other words, one benefit of defending ephemeral stopover territories may be to advance a life history position closer to the breeding life history stage. We assess this by testosterone activity and observations of stopover territoriality. Secondly, we predicted that within individuals, baseline testosterone levels would increase with the number of days spent at stopover (a within-individual seasonal effect), with positive changes in body condition, and with higher refueling rates.

## Results

### Baseline testosterone at stopover (BTS)

We examined the variation in baseline testosterone levels of males during stopover. For this we only included data on males that were passively captured and not manipulated (no playback or hormone injection) and also removed re-captured birds from this analysis. This yielded testosterone samples from 71 passively-captured individuals (53 in 2019 and 18 in 2022). A model for testosterone levels, which tested the effects of year, age, date, sampling time (hour), body condition and territorial male density at capture date was significant (*χ*^2^ = 34.83, df = 6, *P* < 0.001, Table 1). Variables that were significantly associated with higher testosterone levels were body condition, sampling hour and year, while territorial male density had a significant negative effect on testosterone (Table 1). Age and date did not have significant effects on baseline testosterone level of males at stopover (Table 1).

**Table 1:** Model parameters for baseline testosterone (log-transformed) of Subalpine Warbler males (*Curruca iberiae*) during a spring trans-Saharan stopover in Yasmina, Morocco.

Term	Coefficient	SE	*Z*	*P*
Intercept	1.530	0.357	4.30	< 0.001
Body condition	0.047	0.023	2.04	0.041
Territorial male density	−0.011	0.005	−2.11	0.035
Year 2022^a^	−0.352	0.080	−4.39	< 0.001
Age ASY^b^	−0.038	0.054	−0.70	0.482
Julian date	−0.004	0.003	−1.31	0.189
Hour	0.000	0.000	2.23	0.026

^a^Year 2022 is referenced relative to Year 2019.

^b^Age ASY (after-second-year) is referenced relative to the younger age class, after-hatch-year.

### Stopover challenge hypothesis (SCH)

**Fig. 1:**
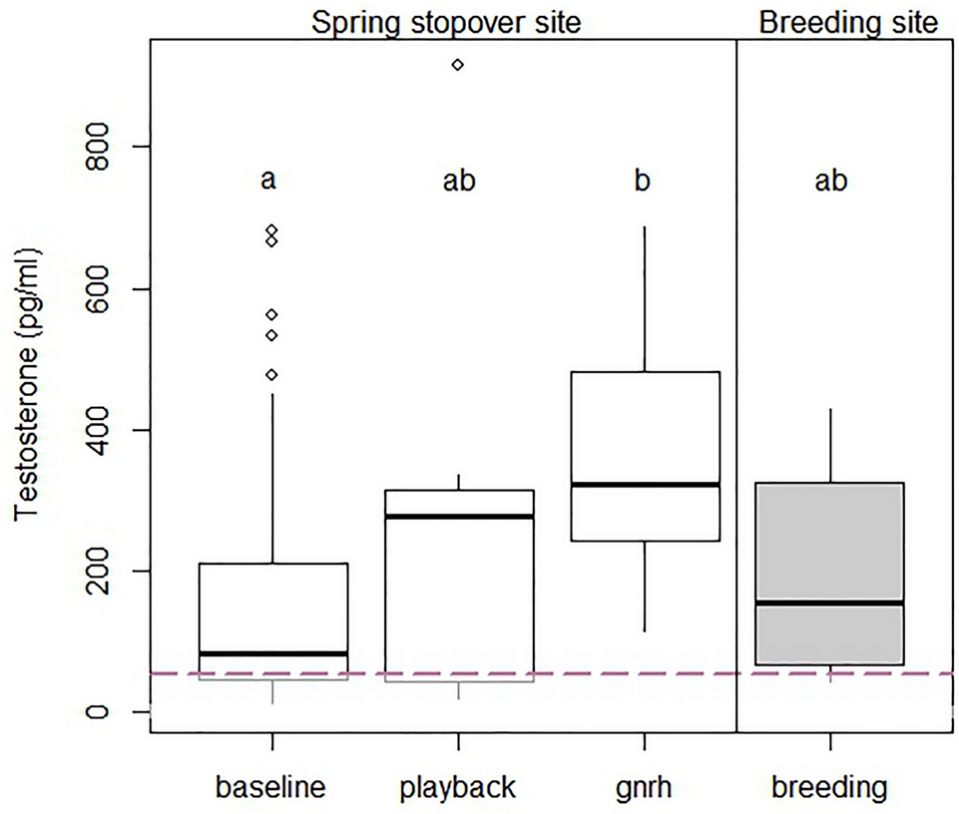
Boxplot illustrating testosterone levels among four groups. Baseline (*N* = 71), playback (*N* = 7) and GnRH (*N* = 7) samples are represented by white boxes and were collected during a trans-Saharan stopover at Yasmina, Morocco. Breeding baseline samples (in gray, *N* = 6)) were collected at a breeding site in the Middle Atlas Mountains in Morocco. The pink dashed line represents the minimum detection value of the testosterone assay kit.

Territorial males subjected to a playback of a conspecific responded aggressively by chipping rapidly, singing and approaching the speaker. This supports that male Subalpine Warblers defend stopover territories^2^. Testosterone levels from these individuals were higher than baseline levels, but not significantly so (*P* > 0.05). Interestingly, GnRH-treated individuals at stopover had comparable testosterone concentrations as the playback individuals (*P* > 0.05) but differed from baseline individuals (*P* = 0.002). Therefore, territory challenge by intrusion appears to increase testosterone levels during migratory stopover approaching a maximal and breeding level (Fig. 1). This result would support the Challenge Hypothesis, however because baseline testosterone did not significantly differ from playback testosterone, this leaves SCH unresolved and thus requires further testing with greater sample sizes.

### Hormonal staging hypothesis (HSH)

#### Comparisons among stopover baseline, playback-induced, pharmacologically stimulated, and breeding baseline testosterone concentrations

We first confirmed that GnRH-treated birds had significantly higher levels of testosterone than did sham controls injected with only saline solution (*t* = −3.943, df = 11.988, *P* = 0.002). We then compared testosterone levels among four groups of male Subalpine Warblers. These were 1) stopover-baseline, 2) stopover-playback, 3) stopover-max (GnRH-treated individuals at stopover) and 4) breeding-baseline. Testosterone levels were significantly different among these groups, with GnRH-treated individuals at stopover containing the highest levels (*χ*^2^ = 12.81 df = 3, *P* = 0.005; Fig. 1). A Peto-Peto pairwise comparison test showed that GnRH-treated individuals had significantly higher testosterone concentrations than stopover-baseline individuals (P < 0.001). This suggests that birds have the capacity to increase testosterone during stopover from baseline and supports gonadal recrudescence during migration (e.g., ref 25). Importantly, stopover baseline, playback and breeding baseline individuals expressed statistically similar testosterone concentrations (*P* > 0.05; Fig. 1).

#### Repeated within-subject testosterone sampling

We performed repeated blood sampling and weighing of 16 individuals. Within-individual sampling intervals ranged from 1–18 days (mean = 8.75 ± 5.702 SD) between captures. There was no relationship between the change in testosterone and fuel deposition rate (i.e., grams per day; p = 0.175). Changes in testosterone levels were not related to stopover duration (i.e., days between sampling, p = 0.782).

**Fig. 2:**
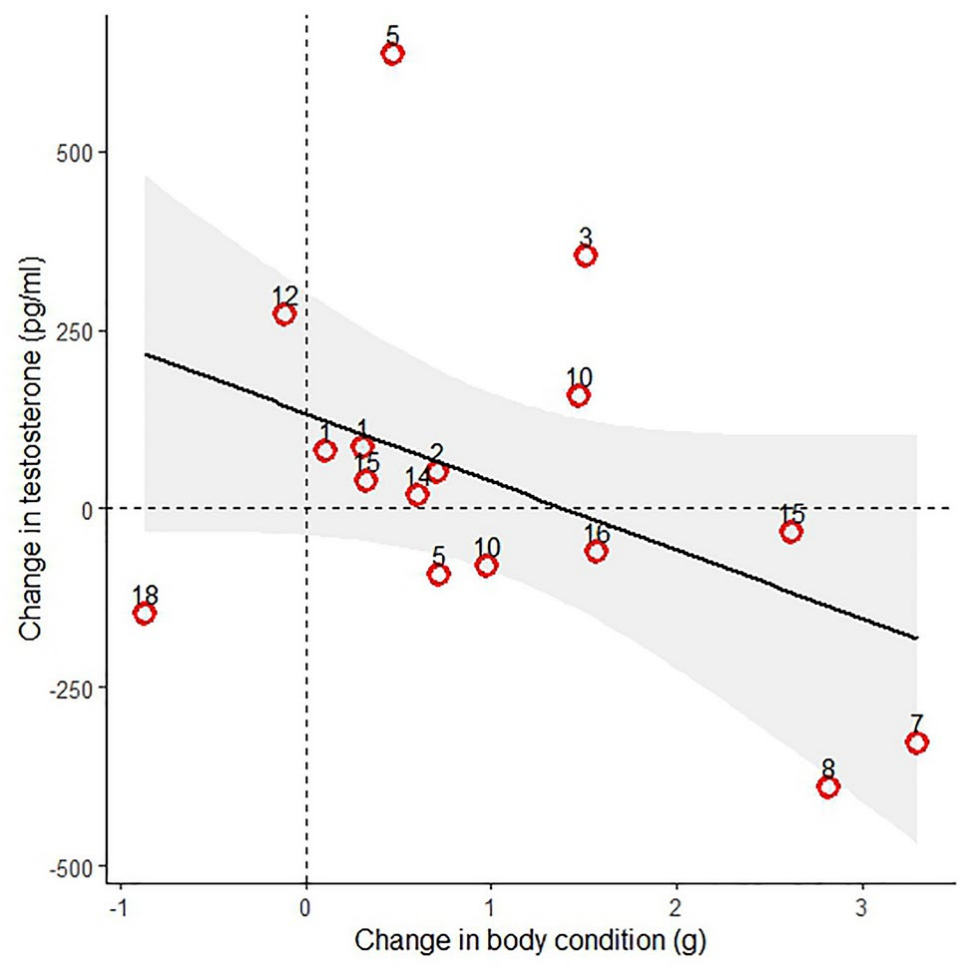
A negative relationship between the change in body condition (scaled mass index in g) and the change in testosterone concentration (pg/ml) between repeated samplings of male Subalpine Warblers (*Curruca iberiae*) during a springtime trans-Saharan stopover in Yasmina, Morocco. The numbers above each point indicate the days between the two samples (which was not significantly related to testosterone). The shaded area shows the 95% confidence interval.

We also calculated the change in testosterone between captures- removing the days between capture from the calculation. The important distinction is that above we looked at fueling rate (grams per day) and here we simply examined overall changes in mass between captures (grams). Here there was a strong negative relationship between the change in testosterone and the change in body condition (adj. *r*^2^ = 0.2986, *F*_1,14_ = 7.387, *P* = 0.016, Fig. 2). Therefore, while fueling rate (grams per day) was not informative in explaining testosterone changes during stopover, the overall change in mass between captures (grams) was significantly negatively correlated with testosterone.

## Discussion

Our study highlights the value of investigating and elucidating how the haphazard dynamics of migration can interact with the hormonal state of birds, potentially impacting the forthcoming stages. Importantly, we evaluated how testosterone in migratory warblers interacts with their energy systems, social contexts, and temporal effects from the environment. This work- placed within a migratory context- may be of particular interest when compared with the more robust field of breeding endocrinology. Between the two fields, we found strong and notable similarities but outstanding differences reflective of the transitional nature of migration.

### Baseline testosterone at stopover (BTS)

During stopover, Subalpine Warblers must find food to replenish energy stores, survive inclement weather, and avoid predation while engaging in fierce territoriality^2,23,24^. Within the BTS component of the study, we describe the baseline hormonal state of birds as they experience the dynamics of stopover from the energetic, social, and temporal points of view.

#### Body condition, migration strategy, and date of sampling influences on testosterone

Baseline testosterone levels were positively associated with body condition. Therefore, birds with higher body condition may also gain advantages associated with higher testosterone levels such as increased aggression, faster and denser muscle regeneration, and higher fat deposition^11,13,25,26^. While body condition is often positively associated with testosterone in breeding taxa, a study of three migratory songbird species sampled during spring stopover did not conclude evidence for this relationship^6^. This suggests that Subalpine Warblers may have a stronger coupling of the energetic and endocrine system at this point in their migration, in comparison to other species from similar studies.

Perhaps this pattern is related to the energy-minimizing migration strategy apparent in Subalpine Warblers. This species avoids alternating bouts of hyperphagia and long-distance flights (i.e., a time-minimizing strategy refs 30 and 2). It also makes prolonged stopovers even with a high body condition- which should predict a short stopover as per time-minimization^27^. Therefore, body condition in the present study likely reflects an individual’s resting state rather than its exercised state. Additionally, many species’ testosterone profiles are likely to become fully developed at the breeding sites under physiologically-rested states, where body condition and testosterone relationships are more obvious^1,28^. For these reasons, the energy-endocrine link in Subalpine Warblers at stopover is more akin to that of a breeding bird, rather than that of time-minimizing migrants. We therefore emphasize the importance of detailing migration strategy when describing the relationship between body condition and life history characteristics.

Within the population sampled during stopover, we did not find an effect of the date of sampling on testosterone levels. This agrees with some studies on migratory songbirds in spring, where the date did not predict testosterone levels (i.e., Covino et al. unpublished but referenced in ref 18). Contrary to this however, Covino et al.^6^found that testosterone increased with progression towards the breeding season, and so did the potential to elevate testosterone^29^. In our case, we suggest that variation of testosterone across the migratory period may be difficult to discern because of the very particular case of stopover territoriality, which introduces testosterone effects from social competition.

#### Territorial male density and testosterone

Importantly, but counter to our prediction, territorial male density had a negative effect on testosterone. We suggest that a high density of occupied territories may represent a saturated stopover site, and therefore, a relatively stable social environment. Indeed, testosterone levels are higher in times of social instability, when territory formation is still occurring^7,16,30–34^. At low densities, the social context may be more unstable, as territory boundaries may be unresolved and therefore still contestable^34^. At higher densities the social context is likely more stable because territories are already established, and so are boundary agreements with neighboring territory holders. This would extend support of the ‘dear enemy’ hypothesis to migratory stopover sites—where territory holders respond less aggressively to other established territory holders than they would to newcomers^35,36^. Alternatively, this pattern may be partially explained by a ‘winner-loser’ effect, whereby successive failed attempts at territory acquisition by floaters depresses testosterone^33^.

Baseline testosterone was higher in 2019 than 2022. This is an unexpected result but it is important to note that yearly variation may normally occur. One possible explanation is that there were more than twice as many Subalpine Warbler males captured in 2019 as in 2022 (172 and 85 respectively, ref 2). Number of individuals can affect density of territories. This difference in testosterone between the two study years agrees with our finding that density of territories is negatively correlated with testosterone.

An important distinction from breeding (or wintering) territoriality is that, in a stopover territorial system, the rate of immigration and emigration is constantly changing, and somewhat haphazardly. Birds crossing the Sahara arrive and leave stopover sites depending on environmental or internal factors^37^, which may lead to rapid and unpredictable changes in population density. The potential for territory acquisition or loss therefore occurs more likely in moments of low territory density. Therefore, increases in testosterone may be more beneficial for acquiring a territory during periods of low density.

Territory establishment during stopover may offer better food acquisition, predation avoidance, shelter from inclement weather, and increased competitive capacity against conspecifics^2,38–41^. Therefore, acute responses in testosterone to dynamic changes in the social environment may be unnecessary or buffered by the relative security of a territory.

### Stopover challenge hypothesis (SCH)

#### Testosterone response to territory intrusion

Following a 10-min song playback, males that endured territory intrusions had higher testosterone levels than baseline levels, but not significantly. However, physiological maximum levels at stopover (induced via GnRH injections) and levels following STI at stopover, were similarly elevated. This supports that prolonged territory defenses at stopover do elicit a facultative response, approaching or reaching the physiological maximum level^7,42^. Therefore, territorial individuals at stopover respond physiologically in a similar way as they would in a breeding context: by raising testosterone levels above baseline and towards a physiological maximum. While this pattern is interesting, we reiterate that it was higher than baseline levels but not statistically, which may be due to low sample sizes. Indeed, further work is needed to confirm the extent of testosterone modulation of ephemeral migratory territories in response to intruders.

Maintaining a breeding territory requires defense against intruders, which may necessitate a ‘facultative’ hormonal response above a certain baseline^8^. Here, we show that this pattern occurs during migratory stopover, as it does for breeding birds. From our observations in the field, territory defenses are usually over within less than 10 s, and this is likely not enough time to trigger such a facultative response in breeding birds^8^. However, A.A.A. observed many prolonged territory disputes between males, including one that lasted 23 min and resulted in a successful defense. Indeed, territory supplantation appears to be rare as the territory owner has many advantages^33^. We suspect that territories at stopover are more likely vacated following the resumed migration of the owner, rather than supplantation.

### Hormonal staging hypothesis (HSH)

#### Stopover and breeding testosterone levels relative to life history transitions

The result that stopover baseline and breeding baseline individuals expressed similar levels of testosterone suggests they are at similar points along a life history axes and that transitioning from one to another may occur quickly and prior to arrival at a breeding site. During stopover there was much variation in baseline testosterone levels. This likely reflects the breadth of positions along a life history gradient (i.e., from non-breeding to breeding) of trans-Saharan migrants. While many individuals at stopover had undetectably low testosterone levels, many others were already expressing similar or higher levels than individuals at the breeding site (Fig. 1). This suggests that for many Subalpine Warbler males, pre-breeding upregulation of baseline testosterone levels occurs at least in part during migration. This result emphasizes previous works detailing that pre-breeding development in migratory birds occurs far before arrival at breeding grounds^18,43,44^.

Migrating birds in the spring express progressively higher testosterone levels with proximity to breeding grounds^6,43,45^. This is likely because gonadal development is controlled by endogenous circannual programs as well as changing photoperiods experienced during the non-breeding and spring migration stages^46–48^. Different from previous studies, we found higher variation in testosterone values collected from a single site. For example, the stopover-baseline testosterone data from Yasmina is comparably variable as the data collected for migrating Garden Warblers (*Sylvia borin*) making stopovers in Tanzania, Ethiopia, Egypt and their breeding grounds in central Europe^43^. This suggests that there is a wide variation in life history positions represented in Yasmina Subalpine Warblers relative to similar species.

Importantly, Subalpine Warblers make an average stopover of 10 days, and seemingly independent of physical condition, while Garden Warbler average 8 h to 2 days, evoking that they are energy-minimizing and time-minimizing species, respectively^2,27,43,49,50^. Migration strategy (time- vs energy-minimizing) influences stopover duration, migration speed, and fuel deposition rate^49^but here, we also suggest that migration strategy may influence the speed of life history transitioning. In silico models showed that migration phenology is an important feature of life history variation, and therefore migration speed may be particularly informative in detailing transitions among stages^51^. Specifically, we would predict that migration speed is correlated with life history transition speed. Therefore, we encourage direct comparisons across species to further explore this potential link.

We suggest that prolonged stopover itself may be beneficial to advancing the breeding phenotype prior to arrival at the breeding site. Arriving at a breeding site with high baseline testosterone has a few key benefits for fitness (e.g., ref 20). For this, we efforted to make repeated samples to learn more about the changes that occur on an individual level during a stopover stay.

#### Fueling rates, body condition, and changes in testosterone within-individuals

Testosterone levels were not associated with fuel deposition rate. Increases in testosterone have been shown to increase pre-migratory fueling^52^. Testosterone may elicit hyperphagia which is associated with faster fueling in migratory birds^26^. Previously, we suspected that Subalpine Warblers were not fast refuelers and that they are likely energy-minimizing migrants. Therefore, this work supports previous assertions about this species as an energy-minimizer.

While the rate of refueling seemed inconsequential, the total change in body condition between first and second capture was negatively correlated with the change in testosterone (see Fig. 2). Again, removing the time component (days) this pattern emerges for our species- an energy-minimizer. We suggest this result encapsulates the energetics and hormonal effects of owning a territory during migratory stopover. Testosterone was generally higher upon the first capture than during the second capture. Upregulations of testosterone may help overcome various challenges, such as dealing with competitors and novel environments^33^. Additionally, migratory flight requires an increase in muscle size which is facilitated by the anabolic effects of testosterone^13,53^. Once a territory is established, a decrease in testosterone may be preferable, especially because chronically high levels may have negative effects on body condition, fitness and survival^15–17^. Accordingly, individuals that lowered testosterone levels during stopover increased their body condition over those that had higher testosterone levels. Therefore, owning a territory may allow an individual to relax its testosterone production during stopover and increase its body condition to ultimately continue migration.

### Ethics approval

All research activities including experiments involving wild birds were approved and permitted by the Moroccan Ministry of Agriculture and the High Commissioner of Water (reference nr. 18/2017; 06/2019; 10/2022). Experiments involving animals were approved and performed in accordance with international, national and institutional regulations and guidelines. This study is reported in accordance with the ‘ARRIVE’ guidelines.

## Conclusion

We show that testosterone is positively related to body condition, is elevated during territory intrusions and that owning a territory may allow for its ‘relaxation’ as body condition is increased or maintained, all during a trans-Saharan stopover. Additionally, testosterone was similar between the migratory and breeding stages. We explicitly note that our migratory samples should not be interpreted as within a reproductive context. However, our breeding-site samples offer a unique insight into a migratory species’ relative position along a life history gradient- peaking at the onset of breeding. Indeed, while stopover represents a life history ‘gray area’, these results suggest that at least within male-male interactions, testosterone behaves similarly to what can be expected within a reproductive context. Nevertheless, we show that classic patterns related to interactions among testosterone, energetics and social dynamics may be applied to the migratory life history stage. We look forward to more studies investigating migratory stopover in a life history context.

## Methods

### Stopover study site

We studied migrating Subalpine Warbler males at Yasmina, Morocco (31.213º N, 03.988º W) from 27 February to 10 May, 2019 and 16 March to 24 April 2022. Females were also captured during this time and info on them can be found in Ref 2. Males of this species establish defined territories in the midst of migration, aggressively defending them and broadcasting songs during prolonged stopovers averaging 10 days^2^. Yasmina is a dry lake bed in the Northern Sahara Desert that abuts a sand dune within a rocky steppe desert containing few scattered patches of vegetated areas. Yasmina functions as a stopover site for many Palearctic migrants in spring and fall^23,54,55^ where birds make use of the Tamarisk trees (*Tamarix* spp*.*) surrounding the dry lake. We captured birds with twenty 12 m-long mist nets operated in a standardized way from sunrise to sunset, except at mid-day to avoid heat stress to netted birds, and also during extremely windy conditions. The elapsed time that birds were left in nets was less than 30 min. We did not monitor individual net time precisely, however this has a minimal influence on androgen levels over this time frame, especially when simply hanging in nets and not directly handled^56^. Handling time was minimized and monitored precisely (detailed in next section).

### Field methods

All Subalpine Warblers were color-banded, aged (as after hatch year or after second year based on plumage), measured for wing chord (mm) and weighed to an accuracy of 0.1 g. In order to estimate stopover-baseline levels of circulating testosterone we took blood samples from birds that were passively captured. Within ten min of removal from the net (mean = 3:27 ± 1:29 SD), we extracted a 70-μl blood sample via brachial venipuncture using a 26-gauge needle and siphoned it into a heparinized capillary tube. The blood sample was transferred into a small Eppendorf tube and placed on ice or immediately centrifuged at 7000 rpm for 10 min to separate the plasma from the red blood cells. We then pipetted the plasma into a small Eppendorf tube, storing it at −20 °C until ready for hormone analysis. If individuals were recaptured, we repeated the bleeding protocol as well as morphometric measures, such as weight. We attempted to re-sample after at least three days, but a couple were bled before this target.

#### Simulated territory intrusions as stopover challenges (SCH)

To test the effect of territory intruders on testosterone levels, in 2022 we coupled simulated territory intrusion (STI) experiments with blood sampling. We made playback tracks from recordings of Subalpine Warblers singing at Yasmina in previous years, and broadcasted them to color-banded individuals on their territories (*N*= 7; detailed in ref 2). Twenty 10-min wav-format tracks were randomly played consisting of alternating bouts of song and silence. Hours to a day before the playback trials, we installed a 6 m closed mist net in the bird’s territory. We then placed a speaker in territories for 10 min, broadcasting songs while observing the behavior of the territory owner to confirm defense. After 10 min of STI, we quickly opened the mist-net, continuing the playback while noting the time elapsed until capture (mean = 3.226 min ± 1.616 SD; modified method from ref^57^). All birds subjected to an STI responded by approaching the speaker, chipping rapidly, or counter-singing (see Ref 2 for behavioral details). When the territory owner was captured, we removed it and took a blood sample. In order to estimate ‘territorial male density’, we spot-mapped for color-banded individuals singing on their territories (detailed in ref 2). Only color-banded individuals seen in the same area on consecutive days were considered territorial and counted for this metric.

#### GnRH-trials

In a random subset of passively captured individuals (*N*= 7), in 2022 we quantified maximum physiological testosterone level. We did this by administering gonadotropin release hormone (GnRH; Bachem Holding Ag, Bubendorf, Switzerland) to stimulate the pituitary, activating gonadal production of testosterone and gauging the sensitivity of the hypothalamic-pituitary–gonadal (HPG) axis^18,58–61^. This has been shown to be a reliable indicator of hormone steroid activity following photo-refractoriness and also used as an indicator of relative life history position^62^. Following methodologies outlined in ref 18, we administered 50 μl doses containing 2.5 μg of GnRH diluted in 1 M phosphate buffer solution. We first cleaned the pectoralis major of the bird with alcohol swabs and injected the muscle with a 50 μl solution using a 29-gauge needle attached to a BD Microfine Syringe (Becton Dickinson, Franklin Lakes, New Jersey, USA). Peak testosterone response to the GnRH injection in small warblers occurs after 30 min^18^. Therefore, after injections, we placed the birds in a cloth bag in a dark, quiet room, and took a blood sample 30 min post injection. In order to control for the potential effect that the injection itself or the 30 min elapsed (and not the GnRH) could affect HPG activity, we administered injections using 50 μl of 1 M phosphate buffer solution as a sham control, and collecting a blood sample after 30 min (*N* = 7).

#### Breeding site sampling

We also sampled a few breeding Subalpine Warblers (*N* = 6) in the Middle Atlas Mountains, Morocco (32.973º N, 05.063º W) between April 30 and May 20 2022. This site is characterized by dense stands of Holm Oak (*Quercus rotundifolia*) of 1–5 m, as well as scattered Juniper (*Juniperus thurifera*) along steep limestone slopes. It is one of the southernmost breeding sites for this species. Here, we erected three 12 m mist nets in breeding territories to target singing individuals. No playback was used, and therefore samples were used as representatives of breeding-baseline testosterone levels. Breeding status was confirmed by the presence of cloacal protuberance and some were observed carrying material for ‘cock-nests’^63^. One such cock-nest was found constructed completely out of sheep’s-wool. Bleeding and storage were as described above.

### Hormone analysis

We quantified testosterone concentration using a commercial enzymatic immunoassay for testosterone (ADI-900–065, Enzo Life Sciences, Farmingdale, NY, USA). Prior to quantification, we extracted hormones using the liquid–liquid extraction method. We added 1 ml of diethyl ether and 150 µl ddH_2_O to the plasma samples, vortexed them, and placed them on a shaker for 30 min. Samples were then centrifuged and freeze-decanted, after which the organic phase was poured into a clean tube and the extraction procedure with residual sample was repeated. After collecting the second organic phase, samples were dried down with N_2_ and re-substituted with 250 μl assay buffer provided by the Enzo kit, and placed overnight at 8 °C. The assay procedures followed manufacturer instructions. All samples were analyzed in duplicates. Detection limit was set at 7 pg/ml. Intra-assay CV% was < 12% and inter-assay CV% was < 4%.

### Data analysis

All data analyses were achieved using program R version 4.3.1^64^. Some of the plasma samples contained testosterone concentrations below the kit lower detection limits, and therefore were assigned as ‘non-detected’. These included 36 samples for the baseline study, two for the STI study and two for the breeding study. All GnRH samples were detected. Importantly for our questions, maintaining the low end (non-detects) of our data may be informative in assessing the nature of the variation of testosterone during migration. While we do not know its value, we know that it is between zero and the lower detection limit, which represents the left-most portion of the natural histogram. Researchers have sometimes dealt with non-detected values by replacing them with a random value, the minimum detection value, or perhaps zero, but these can add unintended structure to the data, artificially modifying the true distribution of the data^65^. Rather, we employed a left-censoring approach with the R package NADA2 and a maximum likelihood estimation of the mean^66^. We used minimum detection values (that varied by sample volume when below the detection limit) to calculate summary statistics such as constructing histograms, quantile plots and obtaining means and standard deviations that integrated both detected values and non-detected intervals. Therefore, while no concrete value is used to replace non-detected samples, the observation that the true value lies between zero and a very low threshold maintains the natural structure of the data and more closely approaches reality than replacement techniques for this application^65^. All hormone data with non-detected values were log-transformed and checked for linearity using the ‘cenCompareQQ’ function and confirmed with a Shapiro-Francia test in NADA2^66^.

### Baseline Testosterone at Stopover (BTS) model

We constructed a left-censored linear model using the ‘cencorreg’ function in NADA2. This was used to evaluate how stopover variables may influence testosterone concentrations in BTS. This model was composed of baseline testosterone concentrations as the response variable, and body condition, age, territorial male density, sampling year, date, and hour as the predictors. Hour of capture was included as testosterone production may vary with circadian rhythms. We estimated body condition by applying a scaled mass index to correct for body size, incorporating a scaling relationship of mass and wing chord^67^.

### Stopover challenge (SCH) and hormonal staging (HSH) models

We used a left-censored linear model to test if testosterone levels differ among the following groups, stopover-baseline, STI, stopover-max (GnRH), and breeding-baseline (second hypothesis). We further used a Peto-Peto test, a post-hoc pairwise-comparison test designed to handle censored values^66,68^, to identify significantly differing groups. This model was used to inform SCH and HSH prediction 1.

Finally, evaluating within-individual testosterone changes during stopover (HSH prediction 2) relies on repeated measures of individuals across days. For this case, we were not able to find an appropriate statistical technique that can incorporate censored values. Therefore, we substituted non-detected values with the volume-specific minimum detection value. We measured changes in mass (corrected to scaled mass index, SMI) to calculate fuel deposition rate, as SMI2-SMI1/days between captures. The change in baseline testosterone between captures (sample2-sample1) was used as a relative index of progression towards the breeding life history stage. We log-transformed testosterone values to achieve a normal distribution and ran a linear model with changes in testosterone between repeated sampling events as the response variable. As covariates, we included days between capture and fuel deposition rate (change in body condition in g/days). Previously, we found that fueling rate was not related to stopover territoriality^2^. Therefore, we ran a separate linear model with testosterone as the response variable and change in body condition between captures as a covariate, omitting the temporal component of days. The difference between the two models is that the first evaluates how fueling rate (grams per day) relates to testosterone, while the second model evaluates how changes between capture (grams) relates to testosterone. For all tests, statistical significance was inferred if p > 0.05.

## Data Availability

Data will be hosted on the Phaidra repository, managed by the University of Veterinary Medicine Vienna and available by request to A.A.A.
